# Non-cirrhotic portal hypertension secondary to cholangiointestinal anastomotic stricture after choledochal cyst excision: a case report

**DOI:** 10.3389/fmed.2023.1149484

**Published:** 2023-05-23

**Authors:** Xu Zhang, Jun Qing Yan, Yan Ying Gao, De Zhao Song, Cheng Lou

**Affiliations:** ^1^Department of Gastroenterology and Hepatology, The Third Central Hospital of Tianjin, Tianjin, China; ^2^Tianjin Key Laboratory of Extracorporeal Life Support for Critical Diseases, Tianjin, China; ^3^Artificial Cell Engineering Technology Research Center, Tianjin, China; ^4^Tianjin Institute of Hepatobiliary Disease, Tianjin, China; ^5^Department of Interventional Radiology, The Third Central Hospital of Tianjin, Tianjin, China; ^6^Hepatobiliary Surgery, The Third Central Hospital of Tianjin, Tianjin, China

**Keywords:** portal hypertension, gastroesophageal varices, choledochal cyst, cholangiointestinal anastomotic stricture, case report

## Abstract

**Background:**

Cystectomy accompanied by biliary system reconstruction is an important treatment option for choledochal cysts, but the risk of post-operative complications is high. The most famous long-term complication is anastomotic stricture, whereas non-cirrhotic portal hypertension secondary to cholangiointestinal anastomotic stricture is rare.

**Case summary:**

Here we report the case of a 33-year-old female patient with a type I choledochal cyst who underwent choledochal cyst excision with Roux-en-Y hepaticojejunostomy. Thirteen years later, the patient presented with severe esophageal and gastric variceal bleeding, splenomegaly, and hypersplenism. Furthermore, cholangiointestinal anastomotic stricture with cholangiectasis was identified on imaging. A pathological examination of the liver suggested intrahepatic cholestasis, but the fibrosis was mild and inconsistent with severe portal hypertension. Therefore, the final diagnosis was portal hypertension secondary to a cholangiointestinal anastomotic stricture after choledochal cyst surgery. Fortunately, the patient recovered well after endoscopic treatment and dilated cholangiointestinal anastomotic stricture.

**Conclusion:**

Choledochal cyst excision with Roux-en-Y hepaticojejunostomy is the recommended standard of care for type I choledochal cysts; however, the long-term risk of cholangiointestinal anastomotic stricture requires consideration. Moreover, cholangiointestinal anastomotic stricture can lead to portal hypertension, and the degree of elevated portal pressure may be inconsistent with the degree of intrahepatic fibrosis.

## Introduction

Choledochal cysts are rare primary lesions of the bile ducts. Most cases occur in infancy and childhood, while ~20% occur in adulthood with an incidence that is 3–4 times higher in women than in men (1, 2). Although an early diagnosis and surgery are the current standard of care, congenital choledochal cysts are prone to secondary complications including biliary calculi, infection, and pancreatitis with a high risk of cancer (3). Inappropriate treatment may result in complex and serious complications such as bile duct inflammation, gallstones, or cancer due to residual cysts and cholangiointestinal anastomotic strictures (4). However, there are few reports on the long-term prognosis of bile duct cysts after surgery. Here, we report the case of a female patient who presented with severe esophageal and gastric variceal bleeding 13 years after choledochal cyst excision with Roux-en-Y hepaticojejunostomy. A cholangiointestinal anastomotic stricture was considered on an imaging examination, while liver pathology suggested intrahepatic cholestasis with mild fibrosis that was inconsistent with the degree of portal hypertension.

## Case presentation

### Chief complaints

A 46-year-old woman was referred to our hospital with melena.

### History of present illness

The patient complained of melena over the previous 2 weeks. Gastroscopy performed successively in our emergency department revealed severe esophageal and gastric varices.

### History of past illness

In 2008, the patient was diagnosed with cholecystolithiasis, cholecystitis, and a choledochal cyst causing persistent right upper abdominal pain for which she underwent cholecystectomy and choledochal cyst excision with Roux-en-Y hepaticojejunostomy. The abdominal pain did not recur after surgery. Abnormal liver function accompanied by slight bile duct dilatation was observed during physical examination in 2015. A liver biopsy revealed an intact hepatic lobule, mild hepatocyte edema with punctate necrosis, slight fibrosis in the portal area with lymphocytic infiltration, and no inflammation at the interface. Thereafter, diammonium glycyrrhizinate and ursodeoxycholic acid were regularly administered. However, her liver function remained abnormal, which manifested with continuous increase of alkaline phosphatase (ALP) and gamma-glutamyl transferase (GGT).

### Personal and family history

The patient had no history of habitual alcohol consumption and no significant history of liver injury–inducing drug exposure. She had no chronic or familial inherited diseases.

### Physical examination

The patient's temperature was 36.7°C, heart rate was 89 bpm, respiratory rate was 17 breaths/min, blood pressure was 125/65 mmHg, and oxygen saturation on room air was 98%. The patient had mild anemia, erythema of the palms, and splenomegaly with a medium-hard texture on percussion.

### Laboratory examinations

Blood examinations indicated moderate anemia (hemoglobin 81 g/L) with low leukocyte (3.24^*^10^9^/L) and platelet counts (61^*^10^9^/L). The prothrombin time and D-dimmer was normal. Liver function tests showed increased levels of several transaminases, especially ALP and GGT, with a normal bilirubin level and a mildly decreased albumin level; the renal function and the blood fat was normal. Liver tests were negative for hepatitis viruses, autoimmune liver diseases, and metabolic liver diseases including hepatolenticular degeneration and hemochromatosis. Exon sequencing revealed a substitution in CYP7B1.

### Imaging examinations

**Figure 1:**
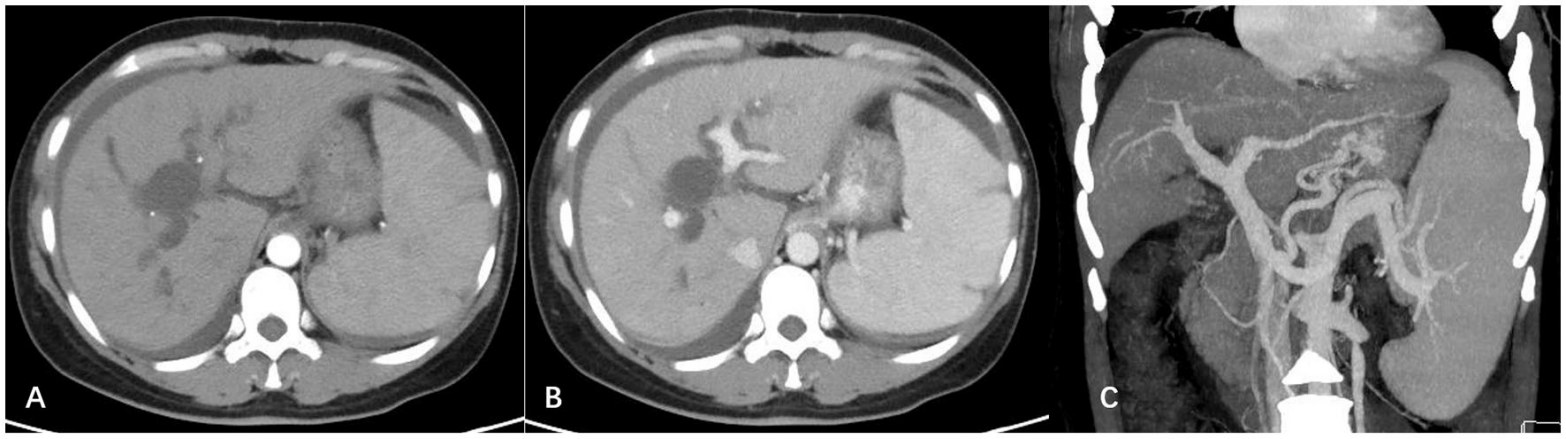
Abdominal enhanced computed tomography images. **(A)** Arterial phase; **(B)** portal vein phase. The outline of the liver was irregular, with a non-uniform density, while the intrahepatic and hilar bile ducts were significantly widened; **(C)** three-dimensional vascular reconstruction of the portal vein system. The portal vein and splenic vein were widened, and multiple tortuous vascular shadows were visible in the lower part of the esophagus and around the fundus of the stomach.

**Figure 2:**
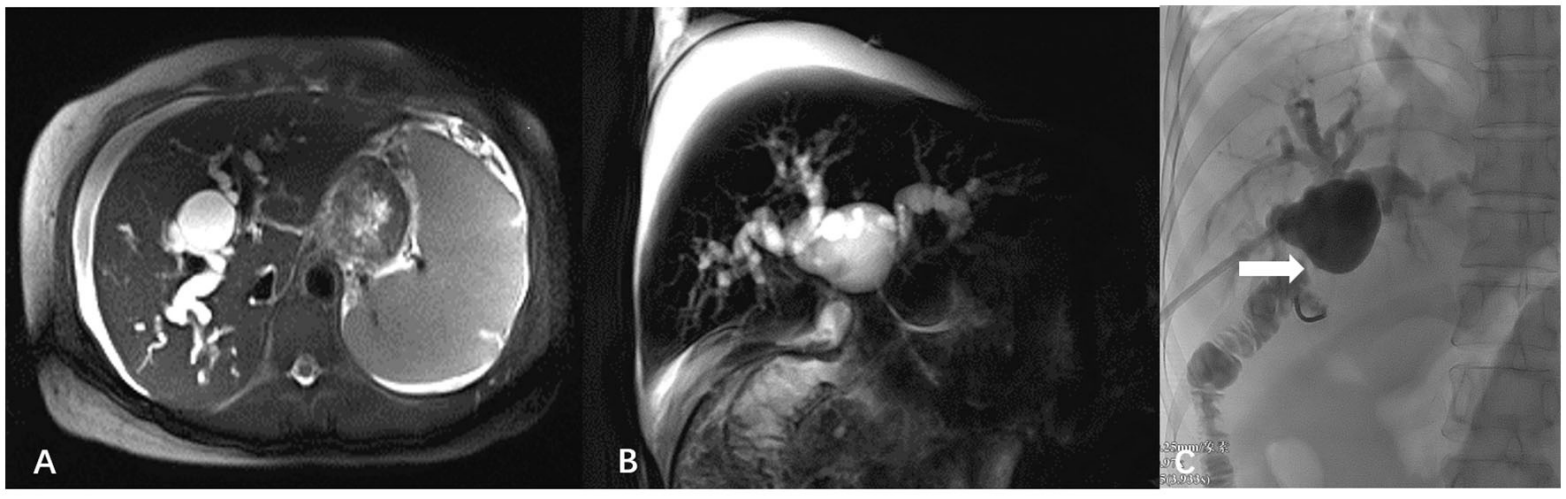
Magnetic resonance cholangiopancreatography images and digital subtraction angiography. **(A)** Axial T2-half-Fourier acquisition single-shot turbo spin-echo (T2-HASTE); **(B)** Sagittal T2-HASTE. The intrahepatic bile duct was significantly widened, cystic dilatation was visible in the hilar bile duct, and the common bile duct was unclear. **(C)** Digital subtraction angiography of cholangiography showed anastomotic stenosis (white arrow).

Enhanced computed tomography of the abdomen showed a relatively regular liver contour, enlarged spleen (splenic length 20.2 cm, thickness 5.7 cm), slightly widened portal vein (diameter 1.4 cm), and varices in the lower esophagus and gastric fundus. The intrahepatic and hilar bile ducts were dilated and the upper segment of the common bile duct was not clearly visualized (Figure 1). Magnetic resonance cholangiopancreatography (MRCP) showed dilated bile ducts in the hepatic region with unclear visualization of the common bile duct (Figure 2). Anastomotic stenosis was confirmed in subsequent digital subtraction angiography of cholangiography (Figure 2).

### Pathological examination

**Figure 3:**
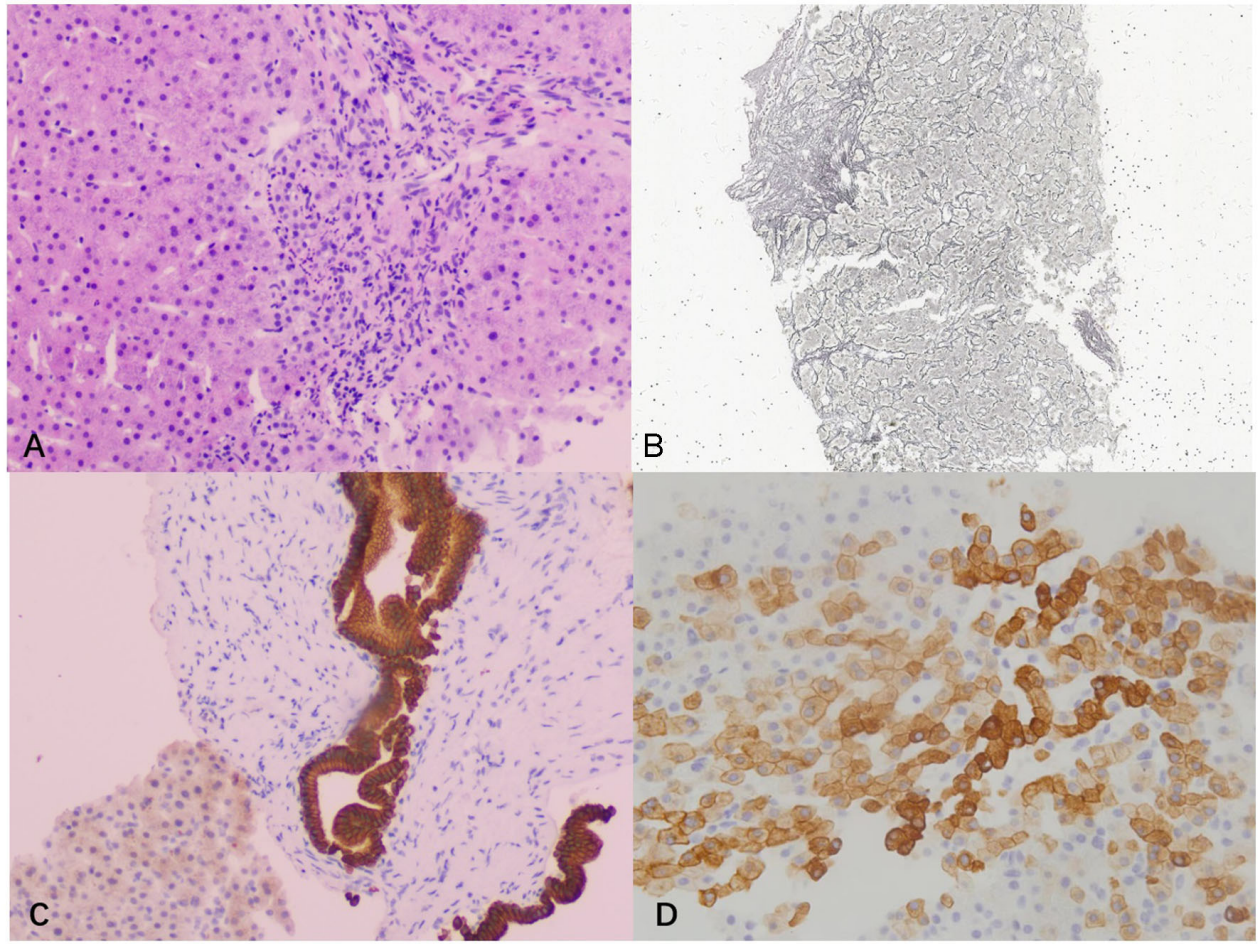
Pathological examination images. **(A)** Hematoxylin and eosin staining showed severe interfacial inflammation (100×); **(B)** silver staining showed mild peri-sinusoidal fibrosis (40×); **(C)** cytokeratin 19 staining showed dilated bile duct (100 ×); **(D)** cytokeratin 7 staining of hepatocytes was positive, indicating chronic cholestasis (200×).

Liver biopsy was performed and the pathology showed essentially intact liver lobules, an orderly liver plate arrangement, and a slightly enlarged hilar region with interfacial inflammation. Mild fibrosis was observed in the interstitium and around the hepatic sinusoid. The epithelium of most small bile ducts showed an irregular pattern, and the hyperplastic portion of the marginal bile ducts was accompanied by mild neutrophil infiltration. Moreover, dilatation of a single bile duct was observed. Immunostaining revealed that some hepatocytes were CK7-positive, indicating chronic jaundice. Therefore, the final pathological diagnosis was chronic mild hepatitis with fibrosis (G3S2) and cholestasis (Figure 3).

## Final diagnosis

The final diagnosis in the present case was non-cirrhotic portal hypertension secondary to a cholangiointestinal anastomotic stricture after choledochal cyst surgery.

## Treatment

**Figure 4:**
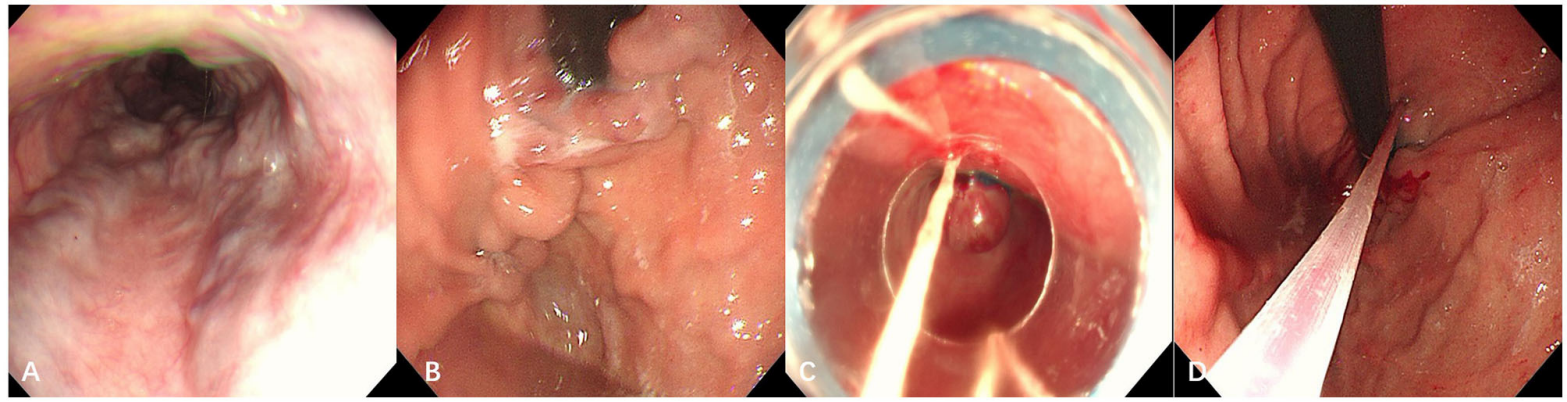
Gastroscopic examination and treatment images taken at our hospital. **(A)** Esophagus; **(B)** gastric fundus. Severe gastroesophageal varices were visible; **(C)** endoscopic varicose vein ligation; **(D)** cyanoacrylate glue injection for gastric varices.

The patient was immediately injected with octreotide to reduce portal venous pressure and stop the bleeding. After the active gastrointestinal bleeding stopped, gastroscopy revealed severe esophageal and gastric varices, Le-smi D3 Rf2, Lg-f D3 Rf2. Ligation of the esophageal varices and sclerotherapy of the gastric varices were performed (Figure 4).

## Outcome and follow-up

The patient's post-operative convalescence was uneventful, with a hospital stay of 7 d. Ten months later, the patient was readmitted to our center for cholangiointestinal anastomotic stricture and underwent percutaneous transhepatic biliary drainage (PTBD) and repeated balloon dilatation. Shortly after the operation, MRCP showed unobstructed drainage from the biliary intestinal anastomosis, near-normal ALP and GGT levels, and relief of varices in the esophagus and fundus of the stomach.

## Discussion

A choledochal cyst, also known as congenital cystic dilatation of the bile duct, is a staged or multiple cystic dilatation of the bile duct that can occur within or outside the liver (5). There are several classification systems for bile duct cysts. The Todani classification system, established by Todani et al. (6) in 1977 based on 37 clinical cases, remains the most widely used classification system for clinical guidance in the surgical treatment of bile duct cysts. Moreover, choledochal cysts can be divided into five types according to the Todani system. Type I is choledochal dilatation, the most common type that accounts for 70–90% of cases. Furthermore, there are three subtypes of type I choledochal cysts: type Ia, overall choledochal dilatation; type Ib, local choledochal dilatation; and type Ic, diffuse fusiform dilatation of the extrahepatic bile duct.

Cyst excision plus cholangiointestinal anastomosis is the standard treatment for bile duct cysts. However, it is extremely difficult to avoid the short-term or long-term complications associated with these procedures despite the surgical techniques and perioperative management have been continuously improved in recent years. The most common long-term biliary complications are intrahepatic bile duct stones, biliary cirrhosis, and biliary intestinal anastomotic stenosis (7). Although the congenital factors including abnormal right artery and type IV bile duct cysts are more likely to induce anastomotic stenosis and intrahepatic bile duct stones (7, 8), we also need to focus more on the technical factors such as incomplete cyst excision, failure of tension-free anastomosis, deficiency of vascular supply, insufficient anastomosis caliber and unresolved hilar or intrahepatic stenosis in type IVA cysts (9). For example, the occurrence of anastomotic stenosis after type I bile duct cyst surgery is mostly due to surgical technique. Although imaging data from this patient's surgery 13 years prior was not available, a type I choledochal cyst was identified based on the description of the choledochal cyst in the surgical record and current images. Moreover, the patient was diagnosed with anastomotic stenosis and bile duct dilatation in the central area of the porta hepatis after surgery, possibly due to incomplete cyst resection and insufficient anastomotic caliber.

Portal hypertension is a rare complication of bile duct cysts. Its mechanism is currently unclear, but it may be related to compression of the portal vein by local cysts, cholestasis-induced liver cirrhosis, and extrahepatic portal vein thrombosis (10–12). The patient had no manifestation of portal hypertension at the time of surgical treatment of the bile duct cyst, whereas ALP and GGT levels were significantly elevated at 7 years after surgery. Autoimmune liver disease was suspected; however, liver biopsy showed only mild inflammation and fibrosis, which did not support the diagnosis. However, long-term liver-protective drugs were ineffective. Six years later, the patient presented with bleeding from ruptured esophagogastric fundic varices, splenomegaly, and hypersplenism. These findings were consistent with portal hypertension. Unexplained portal hypertension with bile duct abnormalities is likely to be diagnosed as congenital hepatic fibrosis with Caroli's disease, which is an inherited bile duct disease caused by a mutation in the PKHD1 gene (13). However, this diagnosis cannot be supported by the results of pathology and genetic sequencing in this case. Imaging examination findings suggested bile–intestinal anastomosis stenosis and intrahepatic bile duct dilation, while liver pathology suggested intrahepatic biliary stasis, which supported the diagnosis of biliary-derived portal hypertension. Therefore, the patient was eventually diagnosed with secondary cholestatic portal hypertension that developed from an anastomotic stricture after bile duct cyst surgery.

This patient suffered from cystic dilatation of hilar bile duct due to bile–intestinal anastomosis stenosis, with a maximum diameter of 5 cm. We believe that the mechanism of portal hypertension secondary to cholangiointestinal anastomotic stricture in this patient is similar with that of portal hypertension resulting from bile duct cysts. Moreover, angiography of the portal system showed widening of the portal vein and establishment of collateral circulation; there was no thrombosis or restrictive stenosis of the portal vein due to the dilated bile duct. Thus, the main cause of portal hypertension in this patient was thought to be cholestatic liver fibrosis. However, pathological examination of the liver revealed a mild degree of fibrosis, a finding that was inconsistent with the degree of elevated portal vein pressure. The increase of portal vein pressure is more likely to develop from compression of portal branches by dilated bile ducts in the hilar region due to obstruction of the biliary system and dilated bile ducts in the Glisson sheath.

The incidence of portal hypertension secondary to biliary cysts is low, and the causes of portal hypertension vary. A uniform treatment strategy is lacking, and reports in the literature are limited to a few case reports of individualized treatment options. The ideal treatment option is choledochal cyst excision combined with structural reconstruction of the biliary tract. Sugandhi et al. (14) demonstrated that liver parenchymal inflammation, cholestasis, and bile duct hyperplasia improved after choledochal cyst resection, whereas short-term improvement of liver fibrosis was not obvious, and long-term prognosis requires further study. Moreover, clinical reports have demonstrated that most patients experience spontaneous resolution of portal hypertension at 6–12 months after surgery (15, 16), and the final clinical prognosis depends mainly on liver function and histology.

However, the ideal surgical solution is often difficult to accomplish because of the presence of collaterals in the portal system. Previous studies reported that cystectomy could be performed successfully after the vascularity of the portal vein region was first reduced by interventional drainage of the cyst, or portal hypertension was relieved by an interhepatic portosystemic stent shunt (11, 17). Interestingly, the portal hypertension in this case was due to stenosis of the bile–intestinal anastomosis after cholecystectomy. Furthermore, there are rare reports in the literature on ruptured bleeding from esophagogastric fundic varices due to portal hypertension or experience with appropriate treatment. To improve the portal hypertension, we would like to reduce the pressure of bile duct dilation and intrahepatic cholestasis by resolving biliary anastomotic stenosis of the patient. However, we found it to be high-risk for the patient because the re-operation requiring lobectomy and more complex biliary anastomosis. Currently, the enteroscopy or percutaneous puncture has been widely used to solve the problem of biliary anastomotic stenosis with good results in clinic (18, 19). In this patient, to reduce the risk of re-bleeding, we first performed endoscopic variceal ligation in combination with sclerotherapy of endoscopic varices, followed by PTBD bile–intestinal anastomosis stenosis dilatation performed twice to improve biliary stasis. Then we found that bile–intestinal anastomosis stenosis was improved dramatically, a significant reduction in esophagogastric fundic varices was observed, and the patient's liver function returned to near-normal levels.

The patient was also diagnosed with a heterozygous *CYP7B1* mutation. *CYP7B1* displays different physiological functions in various tissues, including the liver, reproductive system, and brain. Further, *CYP7B1* is involved in the metabolism of bile acids, and loss of activity is associated with liver failure in children (20). Furthermore, Ye et al. (21) reported several known genetic loci with unique functions in the development and progression of choledochal cysts and suggested that they may be caused by the combined action of multiple polymorphic genes that act independently or cooperatively. However, an association between mutations in the *CYP7B1* locus and choledochal cysts has not been reported, and the relationship between them requires further exploration.

## Conclusion

Cholecystectomy combined with biliary system reconstruction is the standard treatment for choledochal cysts, but it carries the long-term possibility of complications such as bile–intestinal anastomotic stricture and cyst retention. This report describes a rare case of choledochojejunostomy stricture and secondary biliary portal hypertension after choledochal cyst excision with Roux-en-Y hepaticojejunostomy. However, the cholestasis and portal hypertension successfully resolved after the endoscopic treatment of esophageal gastric varices and balloon dilatation of the cholangiointestinal anastomotic stricture. The patient's long-term prognosis was excellent. We believe that this case report provides useful information for such cases in the future.

## Data availability statement

The original contributions presented in the study are included in the article/supplementary material, further inquiries can be directed to the corresponding author.

## Ethics statement

Written informed consent was obtained from the individual(s) for the publication of any potentially identifiable images or data included in this article. Written informed consent was obtained from the participant/patient(s) for the publication of this case report.

## Author contributions

XZ and JY: writing–original draft preparation. YG: gastroscopic treatment and writing–reviewing. DS: performing liver biopsy and percutaneous transhepatic biliary drainage and balloon dilatation. CL: assisted with patient diagnosis and treatment. All authors have read and approve the final manuscript.
